# Material flows accounting for Scotland shows the merits of a circular economy and the folly of territorial carbon reporting

**DOI:** 10.1186/s13021-016-0063-8

**Published:** 2016-09-08

**Authors:** Kimberley Pratt, Michael Lenaghan, Edward T. A. Mitchard

**Affiliations:** 1 Zero Waste Scotland, Moray House, Forthside Way, Stirling, FK8 1QZ UK; 2 School of GeoSciences, University of Edinburgh, Crew Building, The King’s Buildings, Edinburgh, EH9 3FF UK

**Keywords:** Carbon, Scotland, Material consumption, Waste, Circular economy, Climate change, Steel, Neodymium, Lifecycle analysis

## Abstract

**Background:**

It is essential that the human race limits the environmental damage created by our consumption. A realistic pathway to limiting consumption would be to transition to a system where materials are conserved and cycled through the economy as many times as possible and as slowly as possible, greatly reducing the greenhouse gas intensive processes of resource extraction, resource processing and waste management. Material flow analysis (MFA) is a method used to understand how materials are consumed within a nation. In this study, we attempt a MFA for Scotland which links carbon emissions to material consumption using data directly based on the mass of materials used in the Scottish economy. It is the first time such an analysis has been conducted for an economy in its entirety.

**Research aims:**

This study aims to create a detailed material flow account (MFA) for Scotland, compare the environmental impacts and possible policy implications of different future material consumption scenarios and consider two materials, steel and neodymium, in detail.

**Results:**

The model estimated that 11.4 Mg per capita of materials are consumed per year in Scotland, emitting 10.7 Mg CO_2_e per capita in the process, of which, 6.7 Mg CO_2_e per capita falls under territorial carbon accounting. Only the circular economy scenario for 2050 allowed for increases in living standards without increases in carbon emissions and material consumption. This result was mirrored in the steel and neodymium case studies—environmental impacts can be minimised by a national strategy that first reduces use, and then locally reuses materials.

**Conclusions:**

Material consumption accounts for a large proportion of the carbon emissions of Scotland. Strategic dematerialisation, particular of materials such as steel, could support future efforts to reduce environmental impact and meet climate change targets. However, policy makers should consider consumption carbon accounting boundaries, as well as territorial boundaries, if carbon savings are to be maximised. This is because imports and recyclate sent abroad can have significant effect on the carbon emissions from material consumption. We demonstrate that the more circular an economy is, the smaller the difference between global and territorial carbon emissions, and therefore that climate change targets based solely on territorial carbon emissions create perverse incentives. The study also found that there could be areas of economic development which are compatible with environmental aims, based around encouraging reprocessing activities in developed nations.

**Electronic supplementary material:**

The online version of this article (doi:10.1186/s13021-016-0063-8) contains supplementary material, which is available to authorized users.

## Background

### Climate change and the need for dematerialisation

Global anthropogenic greenhouse gas (GHG) emissions released 35 Pg CO_2_ e to the atmosphere in 2014 [1]. These emissions, whether from burning fuels for transport and power, creating buildings, food and products, or heating our water and homes, are ultimately driven by human consumption. It is clear that these emissions must reduce sharply to avoid dangerous climate change and to have any hope of keeping temperature rises below the aspirational 1.5 °C agreed by the United Nations Framework Convention on Climate Change in Paris in 2015. Despite this, there are upwards pressures on emissions from increasing populations and economic growth. These trends force ever increasing levels of consumption of materials—global material extraction has grown by more than 90 % over the past 30 years and is reaching almost 70 billion tonnes annually today [2]. We believe the only realistic route to reducing GHG emissions with an increasing population and living standards is to dematerialise. It is essential that we quickly transition to a system where material use is minimised, and those that are used are conserved and cycled through the economy as many times as possible and as slowly as possible, greatly reducing the GHG-intensive processes of resource extraction, resource processing and waste prevention and management. This requirement is particularly important in nations with highly developed consumer societies, such as Scotland, where materials are consumed in quantities and rates that can never be sustainable.

The environmental need for strategic, global dematerialisation is well documented in academic literature [3, 4]. Material use and productivity are analysed using a technique known as material flow accounts (MFA), standardised by the European Statistical Office (EUROSTAT, Luxembourg) and regularly updated. It is also common to apply economic and environmental factors (even integrating Life Cycle Assessments) to MFA datasets to understand the driving forces and implications of material consumption [5–7].

This study attempts to add to existing knowledge on material flows by linking material specific data to carbon emissions factors for Scotland. It is, the authors believe, the first time such an MFA has been conducted in this detail for a whole nation. This approach offers great flexibility in assessing the carbon emission implications of a circular economy in a real-world context.

There are three aims of this study:

1. To apply detailed material categories to mass and carbon data, creating a detailed material flow account (MFA) for Scotland for the first time.
2. Compare the environmental impacts of different future material consumption scenarios for Scotland, using both territorial and global (consumption) carbon emissions estimates, and consider the possible policy implications of the different scenarios and carbon emission estimates.
3. To compliment the macro, nation level analysis by examining the environmental impacts and policy implications of material consumption scenarios for two materials with economic importance to Scotland, steel and neodymium, in detail.

By conducting this analysis, we hope to show the importance of considering material flows, both at a nation level and at the level of individual materials, to sustainable development.

### The circular economy—a strategic route to dematerialisation

The opportunity to achieve significant emissions reductions while enhancing economic efficiency through dematerialisation has become a popular political concept in recent years. The term “circular economy” (CE) is used by researchers, governments and businesses alike to describe an approach to sustainable development which does not compromise economic growth. In Europe, the European Commission has positioned its forthcoming circular economy package [8] as a central component in both its economic and environmental strategies. Several European nations, including Scotland, have already set out national circular economy plans [9, 10]. Outside of Europe, national governments, including those of the USA, Japan and China are adopting a CE approach [11, 12]. At the same time, there is a rapidly growing body of policy research on the CE. In 2015, the Ellen MacArthur foundation [13], Green Alliance [14] and the Club of Rome [15] all released high-profile studies attempting to quantify the economic and social benefits of a circular economy at a national or European scale. Many businesses have also begun adopting a circular economy approach. For example, the car manufacturer Renault have shown that it is possible to create an CE business model which is internationally competitive: its Espace car is 90 % recyclable [16].

**Fig. 1:**
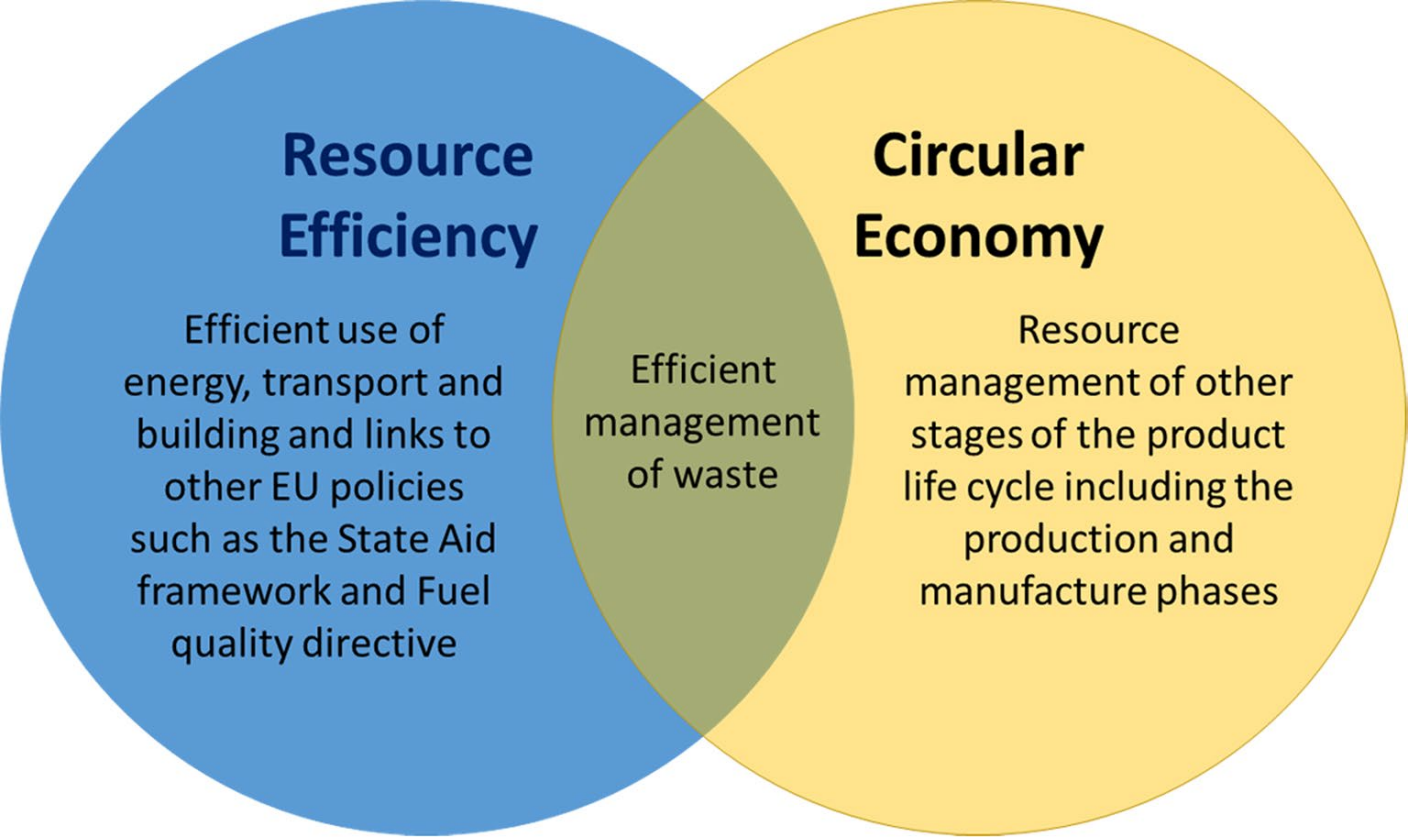
Definitions of resource efficiency and circular economy

This study uses definitions of circular economy and resource efficiency as defined by the European Commission “Roadmap to a Resource Efficient Europe” [17] and illustrated in Fig. 1 below.

According to this approach, the CE aims to “reduce, reuse, recycle, substitute, safeguard, and value” resources across all stages of a product life cycle. Resource Efficiency is a broader term which encompasses improved use of energy, transport and buildings as well as resources. In the context of this study, which focuses on material consumption, the resource efficiency scenario includes the potential savings from improved waste management including waste prevention strategies. The CE scenario considers dematerialisation strategies across all stages of the life cycle, including those relating to production, manufacturing and end of life. An example of a resource efficient material strategy would be if a car manufacturer redesigned their production process so that each car it produced was made of less material. By contrast, in a circular economy scenario, the car manufacturing process may be redesigned so that not only are cars made with less material, they are designed to allow easy remanufacture and repair as well.

### Material flows in Scotland

Our study is based in Scotland, a nation of 5.3 million people within the United Kingdom [18]. It is a developed country with a per capita GDP estimated at $39,642 USD for 2012 [19]. Its economy and environmental footprint are well understood as significant data is collected and research performed by various government bodies including the Scottish Environmental Protection Agency, the UK Office for National Statistics and others. In 2010, Scotland’s total territorial carbon footprint was estimated to be 58,317,631 MgCO_2_e [20] and its consumption carbon footprint 82,175,422 MgCO_2_e [21]. Scotland is typical of many advanced consumer economies as it imports a large proportion of its goods from countries which rely on carbon intensive energy sources and large proportions of potentially recyclable material are disposed of instead. Scotland’s overall recycling rate, which, historically, has been low compared to some European nations is now rising. Its municipal recycling rate was 55.3 % in 2014 [22] compared to a European average of 43 % [23].

**Fig. 2:**
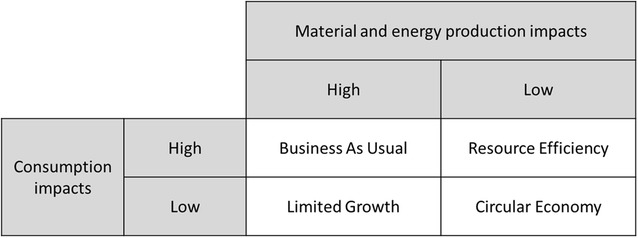
Matrix of the material production and consumption levels considered in the four 2050 scenarios

Scotland has stated its ambition to become a more circular economy [24], in line with its ambitious climate change and waste targets. It aims to reduce GHG emissions by 80 % below 1990 levels by 2050 and recycle 70 % of its waste, sending a maximum 5 % to landfill by 2025. The environmental implications of these ambitions are explored in this study through four scenarios, which extrapolate Scotland’s material use to 2050, as shown in Fig. 2 and described in more detail in the methods. We first consider the whole economy, and then hone in on two materials, the bulk construction product steel and the rare earth material neodymium, as case studies to explore the implications of the above in detail. These materials were selected because of their economic importance, contrasting characteristics, and the availability of good quality data sources.

In the business as usual scenario, production and consumption are assumed to remain at high levels, continuing on from current trends: economic growth is set at 2.2 %; decarbonisation of the electricity grid and recycling trends are assumed to reach their environmental targets. In the resource efficiency scenario, producers, retailers and other businesses reduce production impacts (perhaps due to rising resource prices or legislative pressure) but consumers do not change their behaviours. There is no wider change towards circular economy approaches such as long-life product design, remanufacturing and reuse which may reduce net material consumption as well as efforts to minimise resources used in the production process. In the limited growth scenario, growth is stuck at 0.2 %: businesses fail to adapt their resource use meaning production impacts remain high but consumption is limited by poor economic growth. While the limited growth scenario is extremely undesirable economically and politically, it has been included in this study in order to highlight the correlation between economic growth and emissions that is typical of a linear economy, and thus underscore the benefits of a circular economy in which economic growth and emissions reductions are decoupled and therefore simultaneously attainable. Finally, in the circular economy scenario, it is assumed that both businesses and consumers adapt to a low material impact society. Economic growth is set at 2.2 % but material consumption is assumed to be partly decoupled from this. The basic model assumes 50 % decoupling. This assumption is tested in the sensitivity analysis.

### Steel

Steel is a critical material to any modern economy. Scotland consumed 1.6 Tg of steel in 2012 [25]. Like many developed nations, Scotland was once self-sufficient, but is now entirely reliant on imported steel. If it is recycled at all, this is also done outside of Scotland and the rest of the UK. This has had a marked impact on carbon emissions—when Ravenscraig, the last steel works in Scotland, closed in 1992, national GHG emissions reduced substantially (the Scottish Government estimated that it was responsible for the majority of the 5.3 GgCO_2_e reduction from the Scottish business and industrial process sector between 1990 and 1995) [26]. Although the closure of Ravenscraig helped to reduce territorial emissions, it did not lower Scotland’s global carbon emissions as steel demand did not decrease but was instead imported, often from countries using less efficient methods of steel production. In fact, although this has not been researched directly, it is very likely that Scotland’s carbon emissions resulting from consumption increased as a result of the closure, showing the limitation of territorial-based carbon reporting. This study considers the carbon emissions of creating a modern reprocessing plant in Scotland to recycle steel domestically, compared to sending it abroad.

### Neodymium

Neodymium is a rare earth metal mainly used as permanent magnet in motors for a range of products including wind turbines and electric cars. Global annual production of neodymium is 19 Gg. About 50 % of world mineral reserves of rare earth metals occur in China and 95 % of neodymium production occurs there [27]. Like all rare earth metals, neodymium is difficult to extract and requires large amounts of energy to produce (64 % of neodymium extracted is lost in the production process).

Current use of neodymium for wind turbines in Scotland is low but expected to increase to meet renewable energy targets. It has been estimated that 2.5 Mg neodymium was used in Scottish wind turbines in 2014, and that by 2030, 616 Mg of neodymium are expected to be locked into Scottish wind turbines [28]. Recycling and reuse possibilities for rare earth metals are unproven and likely to be difficult to implement. The environmental savings from recycling will greatly depend on the choice of technology as processing steps could be nearly as energy intensive as virgin production, with estimates for recycling neodymium based on hard disk drives suggesting different methods emitted between 15 and 40 % of the CO_2_ of new extraction [29]. The carbon emissions of three end of life scenarios (landfill, low tech recycling in China and high tech recycling in Scotland) are compared in this study.

### Carbon accounting

The carbon emissions of a circular economy can be quantified in two ways—territorial and consumption based carbon accounting. Territorial or producer-based accounting, centres on the idea of ‘producer responsibility’—emissions produced within a region or country are assigned to that area. This is the standard method used by governments for their international treaty obligations, with most broadly following the methodologies set out under the United Nations Framework Convention on Climate Change (UNFCCC) Kyoto Protocol [30]. In contrast, consumption accounting is based on the idea of ‘consumer responsibility’; it includes all the emissions resulting from consumption, regardless of where those emissions are generated [31]. A focus on territorial accounting, enshrined in international treaties and national accounting processes, has meant that many emissions consumed in the wealthiest nations have been assigned to developing nations instead. Numbers reported by UNFCCC members therefore, if compared to those under a consumption accounting framework, understate the emissions of net importer countries and overstate those of net exporters. More emphasis on consumption accounting would allow the carbon emissions of both producers and consumers to be more clearly understood. In this study we compare and contrast both methods.

## Methods

A desk based model of material flows in Scotland in 2012 (which represented the latest available year for the key datasets) was developed. Four scenarios for 2050 were created: the business as usual, resource efficiency, limited growth and circular economy scenarios. These 2050 scenarios varied the production and consumption of material flows in Scotland, modelling different levels of material circularity to show how these impact on Scotland’s carbon emissions. The scenarios were built on national growth projections [19]. Finally, case studies were developed to explore the impacts of using either consumption or territorial carbon accounting approaches for specific material flow decisions for Scotland.

### The 2012 baseline

The 2012 baseline model combines data on material flows for domestic production, imports and exports to give an estimation of Scotland’s domestic material consumption by material type. This data has been scaled down to Scottish levels based on population from the United Kingdom Her Majesty’s Revenue and Customs (HMRC) data for the imports and exports [32], the Office of National Statistics (ONS) Environmental Accounts [25] and various other sources [33], including personal communications with the Waste and Resources Action Programme (WRAP) for the domestic production data and the Scottish Government regarding economic growth and decarbonisation projections. Data on waste management from SEPA [33] was added to the model to indicate how much waste was managed in and outside of Scotland, as well as the proportions of waste recycled, incinerated and landfilled.

**Fig. 3:**
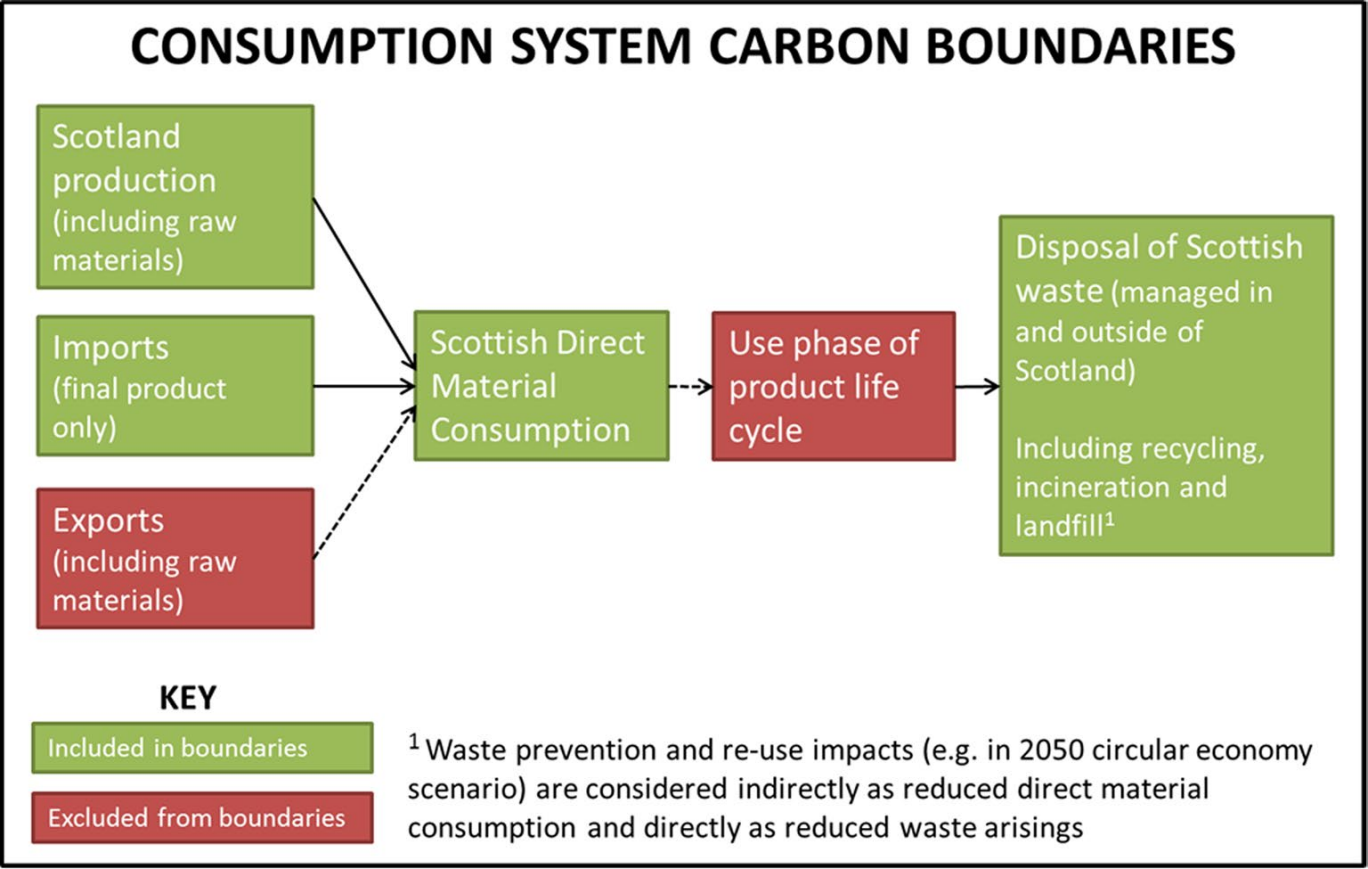
The system boundaries of Scotland’s material and waste flows using consumption carbon accounting boundaries

**Fig. 4:**
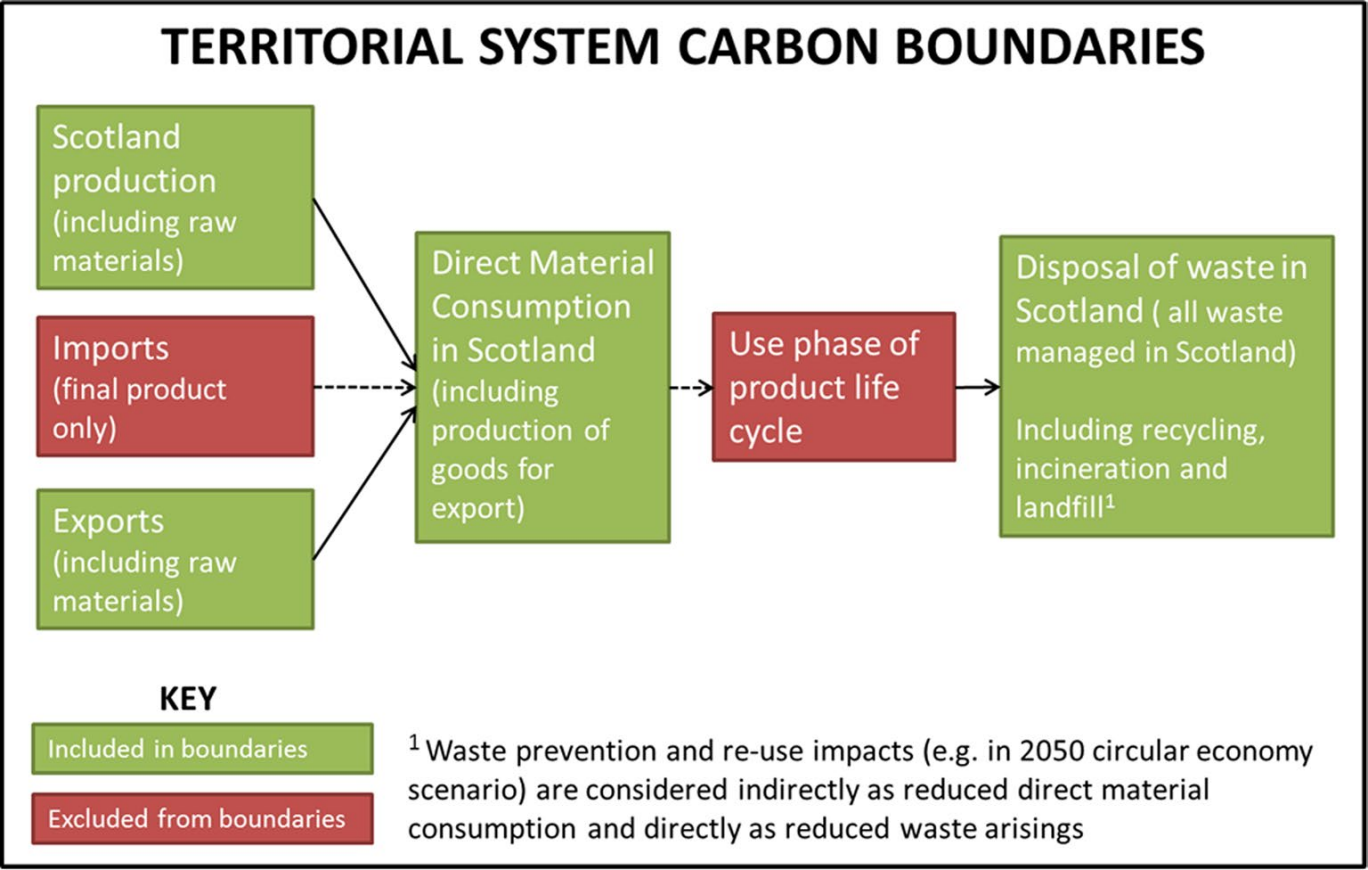
The system boundaries of Scotland’s material and waste flows using territorial carbon accounting boundaries

This tonnage material flows model was then combined with consumption and territorial carbon emissions factors for material production and waste [34]. The consumption model included materials which were consumed by Scotland. So, domestic production, imports and Scottish waste managed inside and outside of Scotland were included, exports and non-Scottish waste managed in Scotland were excluded. The territorial model included all the materials produced and wasted in Scotland, regardless of whether those materials were consumed in Scotland or not. Therefore, emissions from production of goods for export and non-Scottish waste managed in Scotland were included but emissions from the production of imported materials and exported wastes were not. Figures 3 and 4 summarise the main boundaries of the consumption and territorial systems.

### The 2050 scenarios

The 2012 baseline was built on to create four scenarios describing the mass and carbon emissions of material consumption in Scotland in 2050 reflecting different levels of material production and consumption.

Each of the 2050 scenarios are modelled via adjustments to five key drivers: economic growth linked to material consumption; proportion of materials imported; decarbonisation of grid and transport; waste management; and proportion of recyclate exported. The latest Scottish Government report from August 2014 suggests long term growth may be 2.5 %, noting the average independent forecast for Scotland GDP growth in 2014 is also 2.5 %. The assumption of long term growth rates used in this study of 2.2 % is probably slightly conservative but considered appropriate, given the project is forecasting much further into the future than most studies and is bound to include some periods of recession and growth considerably slower than 2.5 %. The peer reviewed WRAP study “Securing the Future” was used as a basis for future growth and imports assumptions [35]. Decarbonisation and waste assumptions were based on meeting Scottish and UK climate change and waste policies (see Additional file 1 for a summary of these policies and how they were used in the model).

### Steel and neodymium case study methodologies

**Fig. 5:**
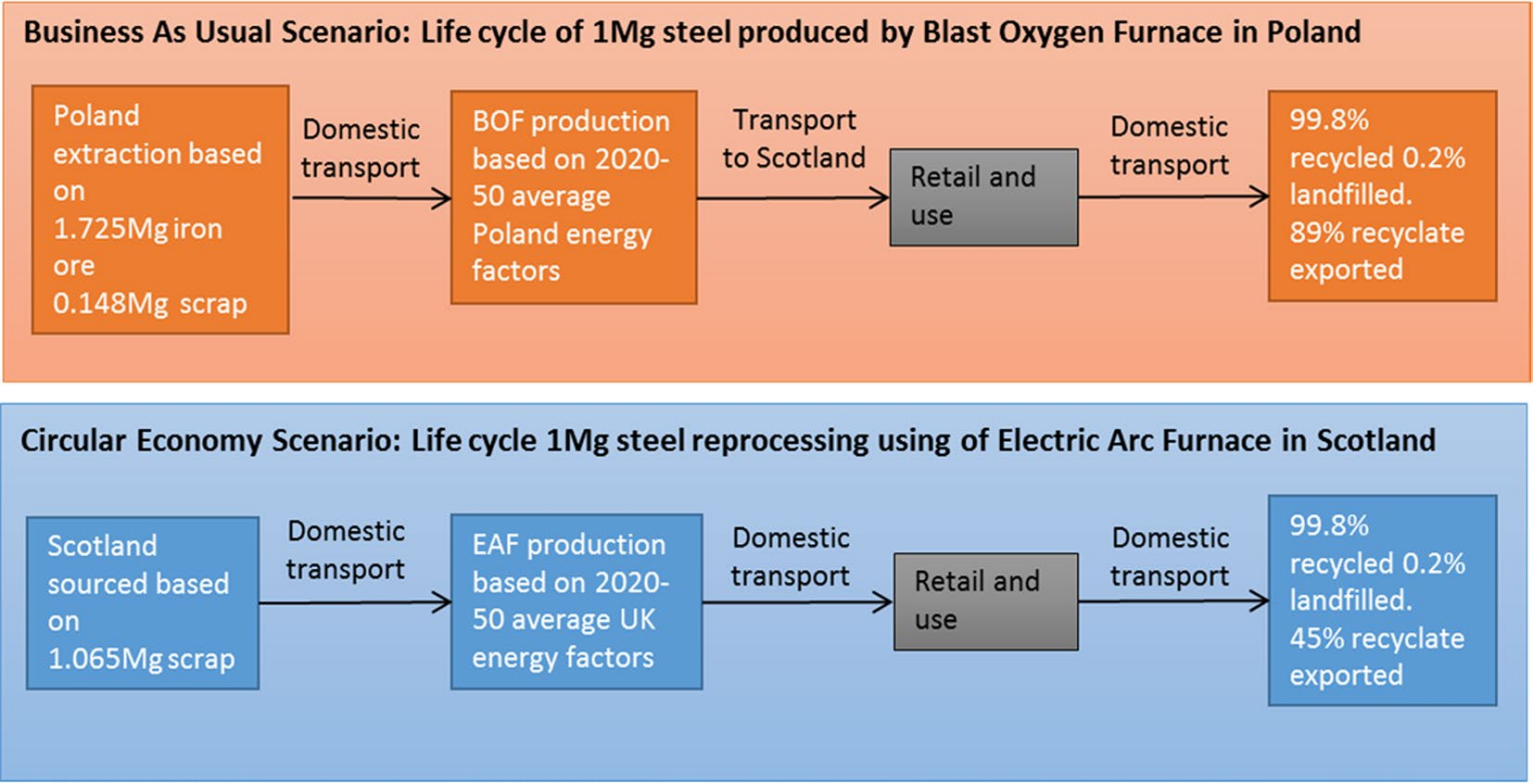
The system boundaries for the steel case study scenarios [33]

The steel data was based on economic and life cycle assessment sources [36]. The system boundaries for the two scenarios are shown in Fig. 5. The BAU scenario estimated the carbon emissions of producing 3 Tg per year of steel (considered to be a small-medium size production plant) for Scottish consumption in a traditional blast oxygen furnace (BOF) plant in Poland (which is taken to be representative of an industrial nation with a carbon intensive energy mix), using 92 % virgin steel, 8 % scrap (BOF plants are not designed to take large quantities of scrap steel). This is compared to a circular economy scenario which considers the carbon emissions of developing an Electric Arc Furnace steel plant which would be operational from 2020 to 2050 in Scotland, and which is capable of reprocessing 3 Tg per year of 100 % scrap steel from Scotland.

**Fig. 6:**
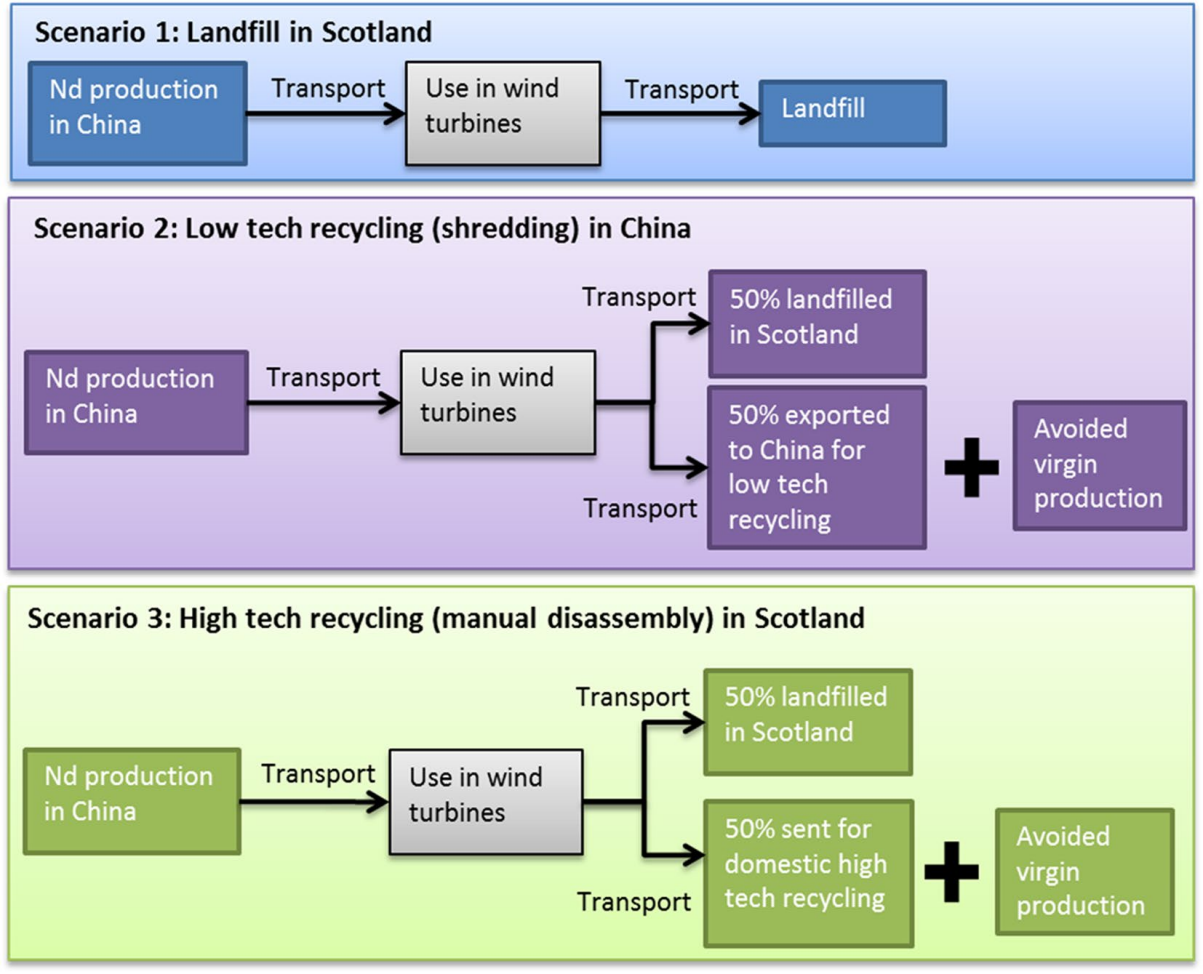
The system boundaries for the neodymium case study scenarios [27]

The neodymium data came from life cycle [27] and wind turbine projections [28] sources. The system boundaries for the three scenarios are shown in Fig. 6.

## Results

The full model and calculations conducted in this study are included as Additional file 1, and can be easily edited and run to allow for the creation of different scenarios or the assessment of the impact of different assumptions. This model should be referred to for exact calculations and definitions of materials, all sources and assumptions. The results in this section are all based on the findings from the basic model. A sensitivity analysis has also been conducted to understand the influence of the main assumptions on the results, this is discussed in “Data quality and sensitivity analysis” section.

### Domestic material consumption and carbon emissions in 2012 and 2050

The mass and carbon emissions of material flows in Scotland were quantified (Tables 1, 2). The domestic material consumption (DMC) was about 60 Tg in 2012 (11.4 Mg per capita), with waste flows about 19 % of DMC (or 2.2 Mg per capita). This results in 56 Tg CO_2_e of consumption carbon emissions, of which only 63 % was captured in an estimate of territorial carbon emissions of 35 Tg CO_2_e.

**Table 1:** The mass and carbon emissions of material flows in Scotland in 2012 (whole nation units)

Indicator	Impact	Unit
Total domestic material consumption	60,436,728	Mg
Total waste arising	11,706,421	Mg
Territorial carbon emissions of total material use	35,455,707	MgCO_2_e
Territorial carbon emissions of waste	285,940	MgCO_2_e
Total territorial carbon emissions	35,741,646	MgCO_2_e
Consumption carbon emissions of domestic material consumption	57,717,771	MgCO_2_e
Consumption carbon emissions of waste	−1,028,215	MgCO_2_e
Total consumption carbon emissions of domestic material consumption	56,689,556	MgCO_2_e

**Table 2:** The mass and carbon emissions of material flows in Scotland in 2012 (per capita)

Material consumption per capita	Impact	Unit
Tonnages consumed	11.40	Mg/per capita
Waste management footprint	2.21	Mg/per capita
Territorial carbon emissions	6.74	MgCO_2_e/per capita
Consumption carbon emissions	10.70	MgCO_2_e/per capita

The model considered 17 material types, shown in Table 3. Carbon factors developed and published by Zero Waste Scotland [34] were used to estimate the emissions associated with producing and disposing of a tonne of each material type. Table 4 shows the most significant materials by three different units of measurement: mass, territorial carbon emissions and consumption carbon emissions.

**Table 3:** Mass and carbon emissions of material flows in Scotland in 2012, by material type

Material type	Domestic material consumption (Mg)	Territorial carbon emissions of material consumption (MgCO_2_ eq)	Consumption carbon emissions of material consumption (MgCO_2_ eq)
Chemical and industrial materials	864,008	676,384	1,145,324
Construction material	14,919,057	1,050,858	1,052,784
Ferrous metal	1,564,815	90,120	4,832,905
Food and plants	6,893,165	15,310,260	21,395,622
Glass	310,272	262,481	278,607
Healthcare equipment	43,479	48,622	77,031
Household goods	294,947	104,938	786,257
Machinery	260,952	40,096	469,191
Minerals	31,939,875	6,353,979	8,456,412
Mixed metals	158,797	460,890	573,926
Non-ferrous metal	335,933	3,905,041	4,359,157
Paper	806,483	349,563	729,267
Plastics	386,895	461,604	1,273,328
Rubber	53,180	77,093	182,786
Textiles	189,510	912,780	4,030,132
Vehicles	518,342	1,271,905	1,772,432
Wood	897,019	315,736	538,539
Total	60,436,728	31,692,351	51,953,702

**Table 4:** Top five most significant materials in the Scottish economy in 2012

Significance	Mass	Territorial carbon emissions	Consumption carbon emissions
1	Minerals	Food and plants	Food and plants
2	Construction material	Minerals	Minerals
3	Food and plants	Non-ferrous metal	Ferrous metal
4	Ferrous metal	Vehicles	Non-ferrous metal
5	Wood	Construction material	Textiles

It can be seen that “Minerals” (a category which includes fossil fuels and other low level raw materials) comes first or second in all three, whereas “Food and plants” have a comparatively low tonnage but higher territorial and consumption carbon emissions. “Ferrous metals” do not enter the top five when considered on a territorial carbon emissions basis, but come third by consumption based emissions. These results show that some high volume materials have comparatively low carbon intensities meaning that mass is not a good indicator of environmental impact. Unfortunately, national recycling rates are nearly always based on mass, limiting their suitability as an environmental indicator.

**Fig. 7:**
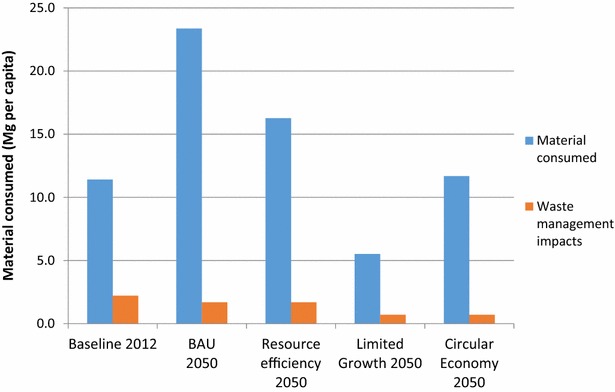
The consumption and waste material flows for Scotland in 2012 and four 2050 scenarios

**Fig. 8:**
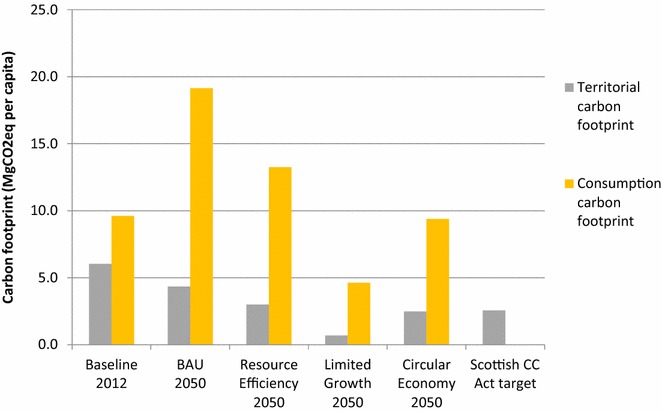
Territorial and consumption carbon emissions for material consumption in Scotland 2012 and four 2050 scenarios

Figures 7 and 8 show the mass and carbon emissions, based on both territorial and consumption carbon accounting, for the 2012 baseline and all 2050 scenarios. Figure 8 also includes the expected per capita carbon footprint for Scottish citizens in 2050 if the Scottish Climate Change Act target of 80 % reduction of 1990 levels is achieved. Only the limited growth and circular economy scenarios have territorial carbon footprints lower than the Act target level. The circular economy scenario has considerably lower material consumption and carbon footprint than the business as usual (BAU) and resource efficiency scenarios and it is approximately equal to the 2012 baseline.

### Steel and neodymium case studies

**Fig. 9:**
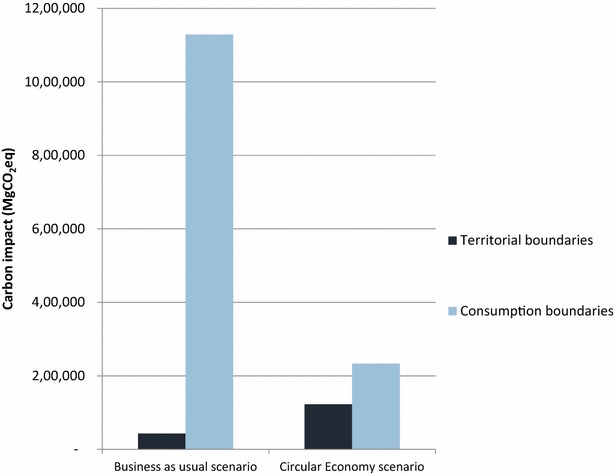
Comparison of the carbon emissions of traditional and circular steel production

The steel case study compares the carbon emissions of producing 3 Tg steel for consumption in Scotland in a traditional, medium sized, processing plant in Poland (a common location for export of Scottish scrap steel) and a largely fossil fuel based electricity mix with reprocessing the same amount of steel using an energy efficient plant in Scotland and using electricity mix based largely on renewable sources (in line with Scottish Government projections for the electricity grid, which is expected to have a carbon intensity of 12 gCO2e/kWh by 2050). Figure 9 shows the results in both territorial and consumption carbon terms. The analysis estimates that 896 GgCO2e can be saved over the 30-year lifetime of the plant in consumption boundaries. If territorial boundaries are used, the carbon emissions in general appears smaller and the traditional steel production process appears to have the smaller impact of the two approaches for Scotland.

The neodymium case study shows that sending all the wind turbines expected to be required by Scotland in 2030 to meet renewable targets to high tech recycling could save 7 GgCO_2_e or 44 % compared to landfill. The carbon emissions from landfill, low tech recycling (abroad) and high tech recycling (in Scotland) depend greatly on whether territorial or consumption boundaries are being considered. For example, the carbon emissions associated with landfill are 3480 times higher in consumption boundary terms than high tech recycling due to the inclusion of raw material processing, which takes place outside Scotland.

**Fig. 10:**
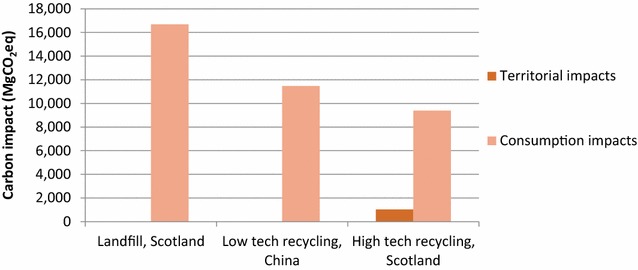
Carbon emissions of end of life options for neodymium in Scottish wind turbines in 2030

The case study shows that for materials used in small quantities but with a disproportionally high carbon cost to extract, efficient, high-tech recycling is highly favourable in terms of carbon emissions using consumption boundaries. However, perversely, using territorial carbon accounting this option appears to increase emissions, as landfill and low-tech recycling in China both have no reported emissions in Scotland, whereas performing recycling in Scotland does produce some emissions within Scottish boundaries. Viewed globally, recycling is clearly the better option: this supports the well-established but rarely used case for consumption carbon accounting in order to align local and global incentives (Fig. 10).

### Data quality and sensitivity analysis

The data available for material flow analysis in Scotland is currently extremely limited. Good material flows data does exist for many countries [37], however, data for the UK is often considered at this level, not the level of its nations, such as Scotland. Since much environmental policy making is devolved to the Scottish level, the lack of Scottish level data makes it difficult for material flow analysis to be effectively embedded into policy decisions. This study does not use any new data sources but attempts to scale UK data sources down to a Scottish level for the first time. This requires assumptions and creates uncertainties where data gaps exist. A data quality assessment and sensitivity analysis were conducted to understand the impact of this on the results (these are available in full in Additional file 1, embedded in the main model file). Key assumptions, such as the degree to which material consumption is decoupled from economic growth in the CE scenario, were varied to understand their influence on the results. The analysis found that the most uncertain datasets were the material flows and carbon factors for domestic production in Scotland. This suggests that caution should be used when interpreting material specific results about domestic production, but changes are unlikely to affect the overall scale of the results.

The results of the sensitivity analysis show that most data changes do not alter the conclusions. The model was most sensitive to changes in material production tonnages (both in the 2012 baseline and 2050 circular economy scenario). Changes to these data and assumptions would change the scale of the results but would not reverse the direction of change. The authors of this paper consider that the reason for this may be that the model itself is relatively simple and robust, with differences between scenarios being large enough that the conclusions are largely insensitive to changes in the input values in realistic ranges.

Three key uncertainties which cannot be tested due to lack of data are: the scale of raw material imports; intra-UK trade impacts of decarbonisation on fossil fuel consumption; and impacts of increased remanufacturing and repair on energy use. Further research is required to improve understanding of these areas and of material flows in Scotland in general.

## Discussion

The analysis demonstrates several interesting observations about material consumption, prioritisation of dematerialisation efforts and the use of territorial and consumption carbon accounting approaches.

Scotland is typical of many developed nations with linear economies—large amounts of material are imported, consumed and then discarded into the waste system. This has a large environmental impact, which is shown in the consumption carbon emissions results of this study but not in the territorial carbon emissions figures. The system of measurement will have a direct impact on any policies to reduce environmental impact from material consumption, such as circular economy efforts. Waste and recycling management in such nations has a net territorial impact but results in a net saving when consumption boundaries are considered. This is because a large proportion of Scotland’s waste is exported for recycling, which has a net carbon benefit when considered across the whole life cycle of a product. Similarly, remanufacturing adds carbon emissions to a nation’s footprint, when considering territorial boundaries (although it should reduce global emissions if logistical and energy efficiencies can be realised). This study shows that as an economy becomes more circular, territorial and consumption carbon accounting approaches begin to converge. It is important for policy makers to understand this and take it into account in decision making—the Paris targets will not be met if governments seek to reduce their emissions by exporting them from both ends of the product life cycle.

### Scenarios

Domestic material consumption impacts vary significantly depending on the different assumptions around economic growth, material production and consumption, and changing import/export ratios. In the BAU scenario, material consumption increases (driven by economic growth) while waste arisings decrease (driven by policy targets) relative to the 2012 baseline. Territorial carbon emissions under BAU also decrease relative to the baseline while consumption carbon emissions increase significantly. Similarly, in the RE scenario, material consumption increases (albeit at a lower rate than in the BAU scenario) and waste arisings decrease, however both territorial and consumption carbon emissions are reduced relative to the 2012 baseline due to partial decoupling of material consumption from economic growth. Material and carbon emissions are lowest in the limited growth scenario, however, the 0.2 % growth rate is politically and socially undesirable. The CE scenario is the only scenario where material consumption and carbon emissions remain stable compared to 2012 levels; though both are higher than in the limited growth scenario, they are considerably lower than in the BAU and RE scenarios despite the high economic growth. In this scenario, supply and demand activities are assumed to become more material efficient through changes such as increased remanufacturing of goods in Scotland and more re-use and repair of products by both businesses and consumers, driving the high levels of decoupling seen. The CE scenario also has as little waste as the limited growth scenario, and far less than the other scenarios or the 2012 baseline.

Based on a purely carbon viewpoint, the limited growth scenario is the most desirable. However, combining significant growth with minimal climate change impact is only possible, from these scenarios, by following a circular economy path. The RE and CE scenarios outperform the BAU on all impacts, suggesting there are no trade-offs of waste for emissions, and that any movement towards these policies will be helpful.

### Steel and neodymium case study results

The steel case study illustrates how different the potential policy conclusions can be when opposing approaches to carbon accounting are considered. Using territorial boundaries, the BAU scenario, seems to have lower carbon emissions than reprocessing steel in Scotland. This is because the production emissions which occur in Poland for this scenario are not attributed to Scotland’s emissions profile. If consumption boundaries are considered, the carbon emissions appear to be much higher (over 1 TgCO_2_e for the BAU scenario) and the CE scenario impacts are lower than the BAU scenario. This is because production emissions are included and production of virgin steel using the Blast Oxygen Furnace system has much greater carbon emissions than reprocessing steel using an electric arc furnace. Also, electricity use in Poland is more carbon intensive because it relies more on fossil fuels, rather than a largely renewable mix in Scotland. The greatest savings between the two scenarios came from the use of reprocessed steel (saving the carbon associated with primary steel production) and the more efficient plant production system, requiring less energy input. The different energy mixes and transport distances are less significant. This highlights the importance of more efficient technology, regardless of location, as well as the impacts associated with production of goods.

The carbon emissions associated with the production of neodymium are considerable—if Scotland consumed, cumulatively, 616 Mg by 2030, driven by renewable energy policies, this could emit over 16 GgCO_2_e. If only territorial emissions are considered, landfill and low tech recycling in China seem to have lower carbon emissions than high tech recycling in Scotland. This is because the production emissions occur outside of Scotland and are therefore not counted under a territorial analysis. However, the consumption carbon analysis shows that the global carbon emissions of neodymium consumption in Scotland can be almost halved if it is disassembled in Scotland. This is because this process allows maximum recovery of neodymium, saving future virgin sources.

Table 5 shows that material consumption is responsible for the majority of Scotland’s carbon emissions, regardless of whether territorial or consumption boundaries are used. This is a new way of considering the carbon emissions of a country and suggests there are opportunities for nations with similar economic profiles to Scotland to reduce both domestic (territorial) and global (consumption) carbon footprints through more circular economies.

**Table 5:** Territorial and consumption carbon footprints for Scotland 2012

Carbon accounting boundary	Material carbon footprint	Total carbon footprint	Material carbon footprint as a proportion of total (%)
Territorial carbon footprint	36 TgCO_2_e (6.7 MgCO_2_e/capita)	53 TgCO_2_e [20]	68
Consumption carbon footprint	57 TgCO_2_e (10.7 MgCO_2_e/capita)	77 TgCO_2_e [21]	74

The materials which contribute most to Scotland’s carbon footprint vary depending on the carbon accounting approach taken. This suggests that policy makers should focus on different material types depending on how they want to maximise their impact (e.g. reducing Scotland tonnage material consumption versus reducing Scotland’s global environmental impact).

The 2050 scenario analysis shows that a CE scenario could save carbon emissions compared to the BAU scenario, in both territorial and consumption terms. Whilst the limited growth scenario shows emissions may be reduced further than even the CE scenario, the economic and social implications of sustained low-growth make it extremely undesirable as an outcome.

The territorial carbon savings of the CE scenario (roughly 11 TgCO_2_e compared to BAU) illustrates how CE strategies can assist Scotland in achieving its ambitious emissions reduction targets in the Climate Change (Scotland) Act 2009 (see Fig. 8) [38]. The Climate Change (Scotland) Act sets in statute a target to reduce Scotland’s territorial GHG emissions by 80 % by 2050 compared to 1990 levels (as well as to consider the impact of consumption emissions, although no formal target is set). Current reporting suggests the Scottish Government is slightly behind it’s reduction plan [39]. Scotland’s delivery plan to reduce emissions [40] does not explicitly consider decarbonisation of materials but many strategies which could make up a circular economy are embedded within the plan, for example decarbonisation of the energy sector with renewable and carbon capture technology. A circular economy strategy would offer additional savings compared to the current delivery plan. An example of this could be to include a recycling plan for rare earth metals used in wind turbines. By incorporating CE strategies for material consumption into climate change reduction delivery plans, Scotland may be better placed to meet its challenging targets.

The case studies on steel and neodymium indicate that, for key materials, a detailed understanding of environmental impacts is required to ensure material consumption is as efficient as possible. There are considerable global carbon savings to be made from steel reprocessing but, if reprocessing is to happen in Scotland, these would be at the expense of territorial climate change targets.

Neodymium is rare and expensive, its production is harmful to human health and highly damaging to the environment. Therefore, despite strong political and technical barriers, the recycling of neodymium should be a priority for any country aiming to maximise its material efficiency. Policy makers should consider the global carbon and material emissions of policies, particularly renewable energy and transport policies, which require carbon intensive rare earth material production. Such impacts are rarely considered now, even in environmental policy making, despite the growing availability of evidence.

## Conclusions

This study applied detailed material categories to mass and carbon data, creating a detailed material flows analysis for Scotland, for the first time. The environmental impact of future material consumption scenarios for Scotland were compared, using both territorial and global (consumption) carbon emissions estimates. In addition, the environmental impacts and policy implications of material consumption scenarios for steel and neodymium in Scotland were examined in detail.

The main findings were:

1. Parametrising a model using this approach can support efforts to prioritise materials for dematerialisation of circular economy efforts, providing useful and relevant estimates of both territorial and consumption carbon footprints. This analysis could be improved by refining material mass data in the future.
2. Adopting a circular economy strategy to material consumption can assist countries in reducing their greenhouse gas emissions and meeting their climate change commitments, without sacrificing economic growth.
3. Certain materials should be prioritised for dematerialisation but the exact materials depend on whether mass, territorial carbon or consumption carbon is considered. This will depend on environmental priorities of nations.
4. There may be areas of economic development, particularly within developed nations with high import levels, which are compatible with broad environmental aims, such as modernising the steel industry to maximise reprocessing and recycling rare earth metals. The case study of expected neodymium demand for wind turbines in Scotland illustrates that material requirements should be considered in environmental policy making, such as policies for meeting renewable energy targets. Consumption accounting over the full economic lifecycle must be considered for renewable energy infrastructure, and in some cases specific investment in local, high-tech recycling may be necessary in order to achieve the desired environmental benefits.
5. Territorial carbon accounting is a poor decision making tool for policy makers aiming to reduce global environmental impact from material consumption. We believe, at the very least, both territorial and consumption figures should be viewed in tandem, and that there is a case for switching to consumption accounting as the primary parameter for international carbon accounting.
6. Future research into the link between materials and products and how these could be represented in analyses of this kind would be a useful step in refining the results. A greater understanding of the carbon emissions associated with raw material production and how to link this to consumption and disposal of the final goods and products is also necessary.

## Additional file

**Additional file 1.** C impact of materials model including the full model, sensitivity analysis and sources used in this study.

## Abbreviations

BAU: business as usual
BOF: blast oxygen furnace
DMC: domestic material consumption
EAF: electric arc furnace
CO_2_e: carbon dioxide equivalents
WRAP: Waste and Resources Action Programme
ZWS: Zero Waste Scotland
